# Development of a Patient-Reported Outcomes Measurement Information System (PROMIS®) short form for measuring physical function in geriatric rehabilitation patients

**DOI:** 10.1007/s11136-020-02506-5

**Published:** 2020-04-21

**Authors:** E. B. Smit, H. Bouwstra, J. C. van der Wouden, C. M. P. M. Hertogh, E. M. Wattel, L. D. Roorda, C. B. Terwee

**Affiliations:** 1 Department of General Practice & Elderly Care Medicine, Amsterdam Public Health, Amsterdam UMC, Vrije Universiteit Amsterdam, Van der Boechorststraat 7 (room B-357), 1081 BT Amsterdam, The Netherlands; 2 Amsterdam Rehabilitation Research Center | Reade, Amsterdam, The Netherlands; 3 Department of Epidemiology and Biostatistics, Amsterdam UMC, Vrije Universiteit Amsterdam, Amsterdam, The Netherlands

**Keywords:** Geriatric rehabilitation, Geriatric patients, Physical function, Patient-reported outcome measure, PROMIS, Psychometrics

## Abstract

**Purpose:**

To develop and test the validity of a Patient-Reported Outcomes Measurement Information System (PROMIS®) short form for measuring physical function of geriatric rehabilitation patients.

**Methods:**

Experts selected items from the Dutch-Flemish PROMIS v1.2 Physical Function (PROMIS-PF) item bank and proposed new items to develop the PROMIS-PF short form for geriatric rehabilitation (PROMIS-PF-GR). Patients evaluated its content validity. Structural validity was assessed by evaluating unidimensionality (confirmatory, exploratory, and bi-factor analyses [criterion: Omega H > 0.80 and ECV > 0.60]), local independence (criterion: residual correlation < 0.20) ,and monotonicity (criterion: H_i_-coefficient ≥ 0.30). Measurement invariance was assessed by evaluating Differential Item Functioning (DIF) between geriatric rehabilitation patients and people from the general population using ordinal logistic regression. Internal consistency was assessed by calculating Cronbach’s alpha (criterion: alpha ≥ 0.70).

**Results:**

Experts selected 24 items from the PROMIS-PF item bank and proposed one new item which was not included in the short form. Patients considered the 24 items relevant and containing essential information. The PROMIS-PF-GR’s psychometric properties were evaluated in 207 patients (mean age ± SD, 80.0 ± 8.3 year; 58% female). The 24 items were found to be sufficiently unidimensional (Omega H = 0.82, ECV = 0.70), locally independent (98.7% item pairs), and monotone (all ≥ 0.32). Five items were flagged for DIF, but their impact on the total score was negligible. Cronbach’s alpha was 0.94.

**Conclusion:**

The PROMIS-PF-GR was developed from the PROMIS-PF and has good content validity, structural validity, measurement invariance, and internal consistency in Dutch geriatric rehabilitation patients. We recommend to confirm the content validity of the PROMIS-PF-GR in other countries.

## Introduction

Patient-Reported Outcome Measures (PROMs) are beneficial for the practice of geriatric rehabilitation because they obtain information about the perceived health directly from the patient and this can potentially enhance patient–physician communication [1, 2]. One of the most important goals of geriatric rehabilitation is to restore or improve physical function, defined as the degree to which a person is able to execute a task or action. Therefore, a PROM measuring physical function would be especially useful for the geriatric rehabilitation setting [3]. Multiple PROMs are available to measure physical function in geriatric patients [4]. However, many of these PROMs have major developmental and psychometric shortcomings, which implies that currently no high-quality PROM is available to measure physical function in this patient group [4]. The absence of such an instrument generates the need to improve existing PROMs or to develop a new high-quality PROM.

The Patient-Reported Outcomes Measurement Information System (PROMIS®) is a psychometrically sound and clinically meaningful measurement system for measuring Patient Reported Outcome (PRO) domains like pain, depression, and physical function [5–7]. PROMIS consists of item banks that measure symptoms and aspects of health-related quality of life. The banks are applicable in a wide range of medical conditions [5]. Each bank consist of a set of items (questions) with responses (answers) that measure the same domain (construct, in this case, physical function) and whose item parameters have been established using Item Response Theory (IRT) analyses [5, 6, 8]. PROMIS scores are reported using a T-score metric (scale or ruler) in which a score of 50 represents the mean score of the general (U.S.) population with a standard deviation (SD) of 10. This makes it possible to compare an individual patient score to the mean score in the general population, facilitating interpretation [9].

An important advantage of PROMIS is that subsets of items of an item bank can be used as so-called short forms, consisting of a fixed subset of highly informative items. Short forms can yield an accurate estimate of a measured domain for a specific patient group [7]. In comparison to the full item bank, they have the advantage of being shorter in length without loss of content validity [7]. Another advantage of short forms is that patients and professionals can specify the content they wish to measure [10]. Related to this, the measure can be tailored to the expected level of the target population at issue on the metric, in this case, the expected low level of physical functioning of geriatric rehabilitation patients.

The PROMIS Physical Function (PF) v1.2 item bank measures self-reported capability and includes lower and upper extremities, central regions, and activities of daily living [11]. It has been translated into Dutch according to PROMIS guidelines and validated in several Dutch clinical samples [12–15]. It consists of 121 items scored on a five point Likert scale, with a higher total score representing a higher level of functioning. Several PROMIS PF short forms have been developed and validated to measure the full range of the PROMIS PF scale [10, 16, 17]. However, geriatric rehabilitation patients are likely to score at the lower and middle end (T-scores < 40) of the physical function scale, and thus, there is no need to ask questions about higher levels of physical function in these patients [18–20]. In addition, some items included in the standard PF short forms are not applicable in the geriatric rehabilitation setting. For example, after total hip replacement, some activities cannot be performed because bending of the hip more than 90 degrees is forbidden. Thus, certain standard items cannot reliably measure level of physical function in these patients. Therefore, we chose to develop a tailored short form instead of validating one of the existing PROMIS PF short forms. The objective of this study was to develop and validate a custom PROMIS PF short form for geriatric rehabilitation patients.

## Methods

### Development

We aimed to select the items from the PROMIS PF item bank that were relevant to geriatric rehabilitation patients. In addition, we considered adding new items that were considered essential for measuring physical function in this group of patients.

We developed the PROMIS-PF-GR short form according to COSMIN guidelines [21]. Firstly, we recruited an expert panel consisting of experienced professionals working in a specialized skilled nursing home facility: two geriatric rehabilitation physicians, two physical therapists, and two occupational therapists. The experts were asked to select items from the v1.2 PROMIS PF item bank and to suggest new items for the PROMIS-PF-GR. One researcher (ES) facilitated the meeting and ensured that all experts had an opportunity to respond and contribute to the item selection and discussion. Another researcher (HB) made notes and summarized the responses of the experts. Both researchers had received training and had experience in qualitative methods. They were non-directive and did not contribute to the discussion. Table 1 summarizes the procedure and provides more details about the expert meeting.

**Table 1:** Selection of the content and items of PROMIs-PF-GR by an expert panel

Prior to the expert consensus meeting
1. The experts received all 121 items of the PF item bank by email and were instructed to review independently the 121 items and judge their usefulness for evaluating physical function of geriatric rehabilitation patients. Experts were specifically instructed to review each individual item and indicate their relevance (i.e., yes/no). Moreover, they were instructed, if necessary, to add new items. The results of these reviews were sent to the primary researcher (ES)
2. The primary researcher (ES) compiled a list of all items that were considered relevant by at least one expert
During the expert consensus meeting
3. The meeting started with an explanation of the goal of the meeting and a presentation of the compiled list
4. Experts were instructed to select 10 to 20 items, as a non-obligatory guideline, which should potentially cover the whole range of physical function of geriatric rehabilitation patients. The experts were also instructed to select and agree on the items, which should be part of the preliminary short form
5. An open discussion followed between the expert panel
6. Once consensus had been reached about the items from the PF item bank that should be included in the preliminary short form, experts were instructed to come up with new items which were not part of the item bank PF and were regarded as essential for measuring physical function in this group of patients

Secondly, we evaluated the content validity (relevance, comprehensiveness, and comprehensibility) of the PROMIS-PF-GR in geriatric rehabilitation patients. It was impractical to organize a patient consensus meeting, because of the frail nature of the patients and logistic problems. Therefore, we interviewed six patients, who were admitted to geriatric rehabilitation, individually. These patients were purposively sampled, aiming at a variety regarding age, gender, and diagnosis groups. The interviews with patients was performed by the same researchers (ES, HB). In addition, we measured the time for completing the PROMIS-PF in these patients. Table 2 summarizes the procedure.

**Table 2:** Evaluation of the content of the preliminary PROMIS-PF-GR by geriatric rehabilitation patients

1. The preliminary PROMIS-PF-GR was presented to the patients and they were asked to comment on the included items, especially commenting on the relevance of the items. In others words, they were asked whether these items represented aspects of physical function that they considered most important during rehabilitation
2. Patients were instructed to fill in the preliminary PROMIS-PF-GR, with final layout, wording of the instructions, items and response options, and to evaluate the formulation the instructions, recall period, and wording of the items and response options, in order to check their comprehensibility
During both steps, patients were also asked to suggest elimination of items, if deemed unnecessary. In addition, patients were asked if there were missing items or information. If necessary they were supported to formulate new items for the PROMIS-PF-GR
3. Finally, the input of all patients was compared. An item was added to the PROMIS-PF-GR, if at least one patient suggested this extra item. An item was removed, if two or more patients suggested its elimination

According to PROMIS policies, a newly created item can definitely be added to a short form if this item improves its psychometric properties [22]. We decide, therefore, to include new items only, if they would meet these conditions.

### Psychometric testing

#### Participants test phase

A first sample, used to study structural validity, measurement invariance, and internal consistency, consisted of geriatric rehabilitation patients from 11 nursing homes in the Netherlands, with a specialized inpatient geriatric rehabilitation ward, included between June 2016 and March 2017. In the Netherlands, frail or older patients who have been admitted to hospital are eligible for geriatric rehabilitation. However, patients with active delirium or dementia are formally not eligible for geriatric rehabilitation. Patients were screened at admission to the ward for study eligibility. Exclusion criteria were the following: decision incompetent patients (as rated by the attending physician), patients who did not master the Dutch language, or patients who did not sign informed consent.

A second sample, used to study measurement invariance, consisted of persons from the Dutch general population. This sample was obtained using an existing internet panel polled by a certified company (Desan Research Solutions) and was representative of the Dutch general population (maximum of 2.5% deviation) with respect to distribution of age (18–40; 40–65; > 65), gender, education (low, middle, high), region (north, east, south, west), and ethnicity (native, first and second generation western immigrant, first and second generation non-western immigrant), based on data from Statistics Netherlands in 2016 [23].

### Procedure

The geriatric rehabilitation patients were measured at the week of admission, three days after the first measurement and at discharge. For the current study, we used the following admission data: demographic and clinical characteristics, presence of a caregiver, comorbidity status, according to the updated Charlson Comorbidity Index (CCI), cognitive functioning, according to the Mini Mental State Examination (MMSE), and functional status, according to the PROMIS-PF-GR. The attending physician rated the CCI. The other information was provided directly by the patients. When patients were not able to complete the PROMIS-PF-GR, a research assistant read each item aloud and the patient verbally expressed his/her answer, which the research assistant filled in. The persons from the general Dutch population were measured at a single time point only.

### Measures

The updated CCI is widely used to measure burden of disease and predict mortality [24, 25]. A score is obtained by assigning a specific weight to each of 12 comorbidity conditions, yielding a maximal score of 24 points, a higher score represents a higher risk of mortality.

The MMSE is a widely used screening test to measure cognitive functioning [26]. The test consists of 11 questions, grouped in 7 categories, representing different cognitive domains or functions, which are added to a total score. The total score ranges from 0 to 30 and a higher score represents a higher level of cognitive functioning.

### Analysis

Missing data were examined for likelihood of systematic or random patterns by analyzing frequencies and patterns of missing data. Descriptive statistics were used to present demographic and clinical characteristics, We used SPSS (IBM SPSS Statistics for Window, version 22.0, Armonk, NY: IBM Corp.) for these analyses.

### Structural validity

We expected that the PROMIS-PF-GR items would load on a single factor as they have mainly been derived from the unidimensional PROMIS-PF item bank. This was first tested with Confirmatory Factor Analysis (CFA) [15, 27, 28]. Model fit was tested by means of the scaled Bentler’s Comparative Fit Index (CFI), the Tucker-Lewis Index (TLI) and the Root Means Square Error of Approximation (RMSEA). An acceptable model fit was defined by the following cut off values: CFI and TLI ≥ 0.95 and RMSEA ≤ 0.08 [28, 29]. A rather liberal criterion of 0.08 was used for the RMSEA because it has been suggested that a commonly used criterion for the RMSEA of 0.06 is too strict for health outcomes [30]. The minimal factor loadings of the items were set at 0.40, and items would be considered for removal in case of lower loadings. We used R Package lavaan (version 0.6-5) for these analyses. We further performed an Exploratory Factor Analysis (EFA) on the polychoric correlation matrix with weighted least square mean and variance (WLSMV) estimation procedures using the R package psych (version 1.7.5) and the ratio of the variance explained by the first compared to the second factor greater than 4 was considered supportive of unidimensionality [8]. Next, the influence of multidimensionality was explored by fitting a bi-factor model and calculating Omega H, Omega total, and explained common variance (ECV). When fitting multidimensional data into a unidimensional model, a high coefficient Omega H (> 0.80) and a high ECV (> 0.60) indicates that the risk of biased parameters model is low [31, 32].

Local dependence was assessed by examining the residual correlation matrix resulting from the single factor CFA mentioned above. Residual correlations greater than 0.20 were considered indicators of possible local dependence [8]. Finally, we assessed monotonicity as a measure of scalability with the R package Mokken. We considered monotonicity acceptable if the scalability coefficients of the items were at least 0.30, and the scalability coefficient for the total scale was at least 0.50 [33].

### Measurement invariance

Measurement invariance was assessed by evaluating Differential Item Functioning (DIF), exploring whether people from different groups, in our study GR patients versus persons from the general population, with the same level of functioning have different probabilities of giving a certain response to an item [34, 35]. We evaluated DIF by a series of ordinal logistic regression models, which model the probability of giving a certain response to an item as a function of the level of physical function (estimated based on all items using a Graded Response Model), a group variable (GR patients versus general population), and the interaction between the level of physical function and the group variable. We used a McFadden’s pseudo *R*^2^ change of at least 2% between the models as a criterion for DIF [36]. Uniform DIF exists when the magnitude of the DIF is consistent across the entire range of function. Non-uniform DIF exists when the magnitude or direction of DIF varies across levels of function. The impact of DIF on the test score was examined by comparing the Test Characteristic Curve (TCC) for all items (ignoring DIF) and the TCC for the DIF items only [37, 38] and visually inspecting the area between the two curves. We used the R package lordif (version 0.3-3) [36] for these analyses.

### Internal consistency

Internal consistency was determined by calculating item-total correlation and Cronbach’s alpha and values of > 0.40 and≥ 0.70, respectively, were considered sufficient [38, 39]. We used SPSS for this analyses.

### Sample size

The DIF analysis required the largest sample of patients. We strived for 200 evaluable patients [40].

### Ethical aspects

The study was approved by the Medical Ethics Review Board of VUmc University Medical Center, Amsterdam, The Netherlands (no. FWA00017598).

## Results

### Development

The expert panel rated 101 out of 121 items of the databank PF as potentially relevant. During the expert meeting ,the professionals reached consensus on a preliminary short form of 26 items. This number of items was considered feasible and sufficient to cover the construct of physical function. The professionals added one new item: “Are you able to ride a bicycle outdoors for at least ten minutes?”. During the patients interviews, one item (item PFC43) out of these 27 items was rated as unclear and was removed. In addition, two items were considered more of less similar (items PFA5 and PFB13), so one of these was removed as well (item PFB13).

The patients considered the remaining 25 items relevant and no essential information was considered missing. Table 3 provides an overview of the 25 items included in psychometric testing.

**Table 3:** Content of the PROMIS-PF-GR with item-total correlation and individual factor loadings

Item number*	Content item	Corrected item-total correlation	Factor loading
PFA5	Does your health now limit you in lifting or carrying groceries?	0.60	0.79
PFA17	Are you able to reach into a high cupboard?	0.63	0.73
PFA23	Are you able to go for a walk of at least 15 min?	0.57	0.84
PFA30	Are you able to step up and down curbs?	0.69	0.78
PFA37	Are you able to stand for short periods of time?	0.66	0.75
PFA45	Are you able to get out of bed into a chair?	0.75	0.86
PFA47	Are you able to pull on trousers?	0.72	0.82
PFA52	Are you able to tie your shoelaces?	0.62	0.77
PFA53	Are you able to run errands and shop?	0.62	0.89
PFA54	Are you able to button your shirt?	0.43	0.57
PFB3	Does your health now limit you in putting a trash bag outside?	0.55	0.79
PFB11	Are you able to wash dishes, pots, and utensils by hand while standing at a sink?	0.71	0.85
PFB17	Are you able to put on and take off your socks?	0.73	0.85
PFB30	Are you able to open a new milk carton?	0.46	0.56
PFB36	Are you able to put on a pullover sweater?	0.55	0.67
PFB48	Does your health now limit you in taking a shower?	0.64	0.68
PFC6r1	Are you able to walk a block (about 100 m) on flat ground?	0.61	0.73
PFC37	Does your health now limit you in climbing one flight of stairs?	0.60	0.81
PFC39	Are you able to stand without losing your balance for several minutes?	0.57	0.67
PFC45r1	Are you able to sit on and get up from the toilet?	0.71	0.80
PFC47	Are you able to be out of bed most of the day?	0.37	0.44
PFC51	Are you able to wipe yourself after using the toilet?	0.60	0.71
PFC52	Are you able to turn from side to side in bed?	0.61	0.68
PFC53	Are you able to get in and out of bed?	0.81	0.91

*Item names in the PROMIS PF item bank

### Psychometric testing

A total of 207 GR patients were included in the study. Table 4 summarizes their characteristics. Most patients were female (58%) and their mean age was 80 years. The main reasons for admission to rehabilitation were the following: stroke (15.5%); elective total joint replacement (15.9%), and trauma, including fractures (27.1%). The mean CCI score was 1.5. The mean MMSE score was 25, indicating relatively low cognitive functioning, and about a quartile of the participants (24%) had a MMSE score ≤ 23, which suggests cognitive dysfunctioning [26]. No item scores were missing.

**Table 4:** Characteristics of the geriatric rehabilitation patients (*n* = 207)

Gender, *n* (%)	
Female	120 (58%)
Male	87 (42%)
Age, mean (SD; range)	80 (8.3; 61–95)
Primary diagnosis, *n* (%)	
Stroke	32 (15.5%)
Elective joint replacements	33 (15.9%)
Trauma	56 (27.1%)
Amputation	2 (1%)
Miscellaneous	84 (40.6%)
Updated Charlson comorbidity index	
Mean (SD; range)	1.5 (1.8; 0–9)
Mini mental state examination, mean	
(SD; range)	25 (4; 9–30)
Mini Mental State Examination ≤ 23, *n* (%)	44 (23.8%)
PROMIS-PF-GR T-score	
Mean (SD; range)*	26 (8;11–51)
Caregiver presence, *n* (%)	
Yes	112 (54%)
No	91 (44%)
Missing	4 (2%)

*n* sample size, *PROMIS-PF-GR* PROMIS Physical Function short form Geriatric Rehabilitation, *SD *standard deviations

*T-scores are based on US item parameters

### Structural validity and internal consistency

The fit indices of the 25 items indicated good model fit regarding CFI (0.95) and TLI (0.95), while the RMSEA (0.08, 95%CI 0.074–0.090) indicated marginally good fit. All item factor loadings were > 0.40 (range: 0.44–0.91). EFA first factor eigenvalue was 12.4, second factor was 2.05, and ratio was 6.07. Bi-factor analysis: Omega H 0.82, Omega total 0.95, and ECV 0.70. Out of 300 unique item pairs, 17 pairs (5.6%) had a residual correlation > 0.20. Monotonicity: H items 0.33–0.65 and H scale 0.52. Cronbach alpha was 0.94. Two items had an item-total correlation lower than 0.40: items PFNL01 (0.35) and PFC47 (0.37). Given this low item-total correlation, it was decided to remove item PFNL01 as this was a new created item that did not seem to improve the psychometric properties of the short form. Item PFC47 was kept because, according to the experts, it has good face validity to determine lower physical function in geriatric rehabilitation patients, the factor loading was also sufficient.

As a consequence the analyses were repeated on the remaining 24 items. The CFA fit indices remained sufficient for CFI (0.95) and TLI (0.95), while RMSEA became marginally unacceptable 0.09 (90% CI 0.077–0.094). EFA first factor eigenvalue 12.5, second factor 1.90, ratio 6.56. Bi-factor analysis: Omega H 0.83, Omega total 0.95, and ECV 0.71. Out of 300 unique item pairs, nine (2.7%) pairs had negative (≤0.20) and four (1.3%) pairs (PFB36-PFB30, PFA37-PFC39, PFA54-PFB30, PFA54-PFB36) had positive residual correlations (0.245–0.332). Monotonicity: H items 0.32–0.65 and H scale 0.52. Finally, factor loading, item-total correlations, and Cronbach alphas did not change considerably for the 24 items model. In conclusion, data showed that the 24 item PROMIS-PF-GR has sufficient unidimensionality. The final PROMIS-PF-GR, therefore, consisted of 24 items and the subsequent analyses were conducted on this version. The time needed for completing the PROMIS-PF-GR ranged between four and seven minutes.

### Measurement invariance

The general population sample that was used for DIF testing consisted of 1310 people, 53% was female and their mean age was 51 (range 19–87). Five out of the 24 items were flagged for uniform DIF for GR patients versus general population: PFA37, PFA53, PFA54, PFB36, and PFC45r1. The McFadden’s pseudo *R*^2^ values for change of these five items ranged from 0.023 to 0.029 which was just above the criterion of 0.02. Four out of the five (PFA37, PFA54, PFB36, PFC45r1) TCCs were slightly higher in the GR patients than in the general population sample, indicating that GR patients endorsed higher response categories at the same level of physical function. The impact of DIF on the total score was negligible.

### T-scores

The mean T-scores of the PROMIS-PF-GR was 26 with a range of 11 to 51. The mean T-score metric of the general (U.S.) population is 50 with a standard deviation (SD) of 10, a score between 30 and 40 represents moderate limitations (70% of the patients in this sample) and below 30 severe limitations (23% of the patients in this sample) in physical function [41]. Thus, the scores of the geriatric rehabilitation patients ranged from average to more than two SDs below average and 93% of the sample had a T-score that is considered moderate or less.

## Discussion

We developed and tested content validity, structural validity, measurement invariance, and internal consistency of the PROMIS-PF-GR, a PROM intended to measure self-reported physical function in GR patients. It was developed based on the existing validated PROMIS PF item bank, with involvement of experienced professionals and geriatric patients, and contains 24 items. The content validity and structural validity were considered sufficient. Five items from the PROMIS-PF-GR were flagged for uniform DIF, but their impact on the total score was negligible. The internal consistency was sufficient. Only item PFC47 had a corrected item-total correlation lower than 0.40, which suggest that this item does not correlate well with the other items of the PROMIS-PF-GR. However, we decided to keep this item because the experts agreed that this item has good face validity for measuring lower physical function.

The expert group suggested one new item (PFNL01) which examines the ability to ride a bicycle outside. In the Netherlands, riding a bicycle is a culturally relevant physical activity and adding this item would contribute to the content validity. From an international perspective, however, adding this item might be less appropriate as riding a bike is not relevant in all cultural contexts. Moreover, as this item is not included in the original PROMIS PF item bank and our sample size was too small to estimate the its parameters, it is not possible to calculate a T-score including this item. Furthermore, the item showed a low item-total correlation. We concluded, therefore, that we do not have evidence that adding this item improves the psychometric properties of the short form, and we decided to remove the item from the current version of the PROMIS-PF-GR. We recommend further research in a larger sample, to evaluate whether adding an item on riding a bicycle would improve the psychometric properties of the PROMIS-PF-GR.

An important reason for developing a custom short form instead of validating an existing PROMIS short form was the expected lower T-score range of geriatric rehabilitation patients. The T-scores of the PROMIS-PF-GR in this study ranged from 11 to 51, with a mean of 26. According to PROMIS guidelines a T-score below 30 is representative for severe limitations in physical function, which supports our hypothesis that geriatric rehabilitation patients are likely to score at the lower and middle end of the physical function scale.In comparison, the possible range of T-scores for the standard PROMIS-PF 20a short form is 9 to 63, with an expected mean of 50 in the general population [41, 42]. This shows better targeting of the PROMIS-PF-GR than the standard PROMIS-PF 20a for geriatric patients. In addition, the content of the PROMIS-PF-GR is considered more relevant for geriatric patients as compared to the standard PROMIS-PF short forms, which contain some inappropriate items for this population.

An unexpected finding of this study was the relatively low cognitive functioning of the participants in the first week of admission, as indicated by the MMSE score. It has been shown that it is hard for persons with cognitive impairments to understand questions and choose response options [43, 44]. Kramer and Schwartz recently proposed specific recommendations for the use of PROMs in the presences of cognitive impairment on content, layout and administration [45]. We believe that we have to a large extent complied with these recommendations. The PROMIS-PF-GR was administered as a paper version instead of using a computer or device and in case patients still were not able to complete this paper version, a research assistant read each item aloud and the patient verbally expressed his/her answer, which the research assistant filled in. In conclusion, our study results suggest that it is possible to measure self-reported physical function even in the presence of low cognitive functioning. This is in line with findings of Tatsuoka et al. who found that cognitive status generally did not have a significant effect on PROMIS Physical Function scores [46].

The current study showed that the PROMIS-PF-GR had sufficient unidimensionality bi-factor analysis Omega H 0.82, ECV 0.70) This is in accordance with the original PROMIS PF developmental study as well as with three validation studies of this item bank in the Netherlands [11–14]. Unidimensionality is an important prerequisite to enable IRT-based scoring and this also makes it possible to compare T-scores of our custom short form to the original PROMIS item bank and other shorts forms. We found four item pairs (1.3%) with positive residual correlations > 0.20, indicating some degree of local dependence. This is likely due to similarities in wording (for example, button a shirt and put on a sweater, two items about standing), which are not affecting the measurement of the construct of physical function. Moreover, the impact on the total score will probably be small. Nine item pairs had negative residual correlations, indicating some degree of multidimensionality, which is in line with the relatively high RMSEA (0.09). However, the scale was considered ‘unidimensional enough’ based on the bi-factor results. There were no problems with monotonicity. We, therefore, conclude that it is possible to create custom short forms with good measurement properties from an existing IRT-based item bank.

Several custom PROMIS PF short forms have been developed recently and tested in clinical groups and older people. The structural validity of a custom 16-item PROMIS PF short form was determined with CFA in a group of patients with cancer (*n* = 5318). This study found comparable results for the CFI (0.98);however, the RMSEA (0.11) showed suboptimal fit [16]. Another study developed multiple PROMIS PF short forms and evaluated the validity in comparison to traditional legacy instruments in “normal” aging people over 65 years and those with osteoarthritis and rheumatoid arthritis (RA) [17]. This study concluded that these short forms outperformed legacy instruments and recommended their use instead of the legacy instruments. Oude Voshaar et al. developed a 20-item PROMIS short form by selecting items that corresponded to the ICF core set for RA [10]. They concluded that the short form reflected the physical function domain for patients with RA and the measurement precision surpassed that of other physical function instruments [10]. The study did not address the structural validity of the short form.

One important advantage of PROMIS is that subsets of items of an item bank can be used either as short forms or as Computerized Adaptive Tests (CATs). CATs use an algorithm that selects the most informative items from the item bank, based on the individual’s responses to previously administered items. CATs have the advantage of measuring the ability level of a person with a minimal number of items without loss of measurement precision [47, 48]. Important disadvantages of CATs are their need for information technology and the costs related to this [14]. Although CATs might be superior to short forms, in terms of feasibility, reliability, and responsiveness, we decided to develop a short form, because of technical difficulties and cost of using CATs in geriatric rehabilitation [12, 13, 47].

One of the strengths of this study is that the PROMIS-PF-GR was developed based on items of the PROMIS-PF item bank. PROMIS item banks have been calibrated, validated, and have well-established item parameters [5, 6, 8]. As a consequence, total scores of the PROMIS-PF-GR can be converted into T-scores, which are anchored to the general population, which facilitate the interpretability of the PROMIS-PF-GR scores and enable comparisons with PROMIS-PF item bank scores. Another strength was that the PROMIS-PF-GR was tested in multiple geriatric rehabilitation wards across the country. There are also some limitations to this study. Firstly and most importantly, because of the frail nature of the patients, we were not able to hold a patient consensus meeting. Still, we involved individual geriatric patients in the developmental phase and the input of these patients was essential in the final composition of the PROMIS-PF-GR. Secondly, we did not include existing questionnaires as a potential source for the development of new items for the short form. However, we felt that existing questionnaires were already sufficiently screened by the developers of the original PF item bank [49]. Thirdly, the PROMIS-PF-GR contains 24 items, which can be considered too long for a single domain measure in clinical practice settings. Still, the PROMIS-PF-GR contains items which are considered relevant for geriatric rehabilitation by both patients and experts. The time for completing the PROMIS-PF-GR ranged between four and seven minutes which can be regarded as acceptable.

Before implementing the PROMIS-PF-GR into the field of geriatric rehabilitation, future studies should determine other important measurement properties of the PROMIS-PF-GR, like test–retest reliability, responsiveness, and its clinical interpretability. In countries where the PROMIS-PF item bank has already been translated, the PROMIS-PF-GR items can be created from the language-specific PROMIS-PF item bank. We recommend to confirm the content validity of the PROMIS-PF-GR in countries outside the Netherlands.

## Conclusion

The PROMIS-PF-GR is a new IRT-based PROM consisting of 24 items to measure self-reported physical function in geriatric rehabilitation patients. It has been developed as a short form from the PROMIS Physical Function item bank with the involvement of both experienced professionals and geriatric rehabilitation patients. It has sufficient content validity, structural validity, measurement invariance, and internal constancy, and its T-score can be compared to short forms and CATs from the same item bank.
